# Trend of myopia through different interventions from 2010 to 2050: Findings from Eastern Chinese student surveillance study

**DOI:** 10.3389/fmed.2022.1069649

**Published:** 2023-01-18

**Authors:** Xiyan Zhang, Yonlin Zhou, Yan Wang, Wei Du, Jie Yang

**Affiliations:** ^1^Department of Child and Adolescent Health Promotion, Jiangsu Provincial Center for Disease Control and Prevention, Nanjing, China; ^2^School of Public Health, Nanjing Medical University, Nanjing, China; ^3^School of Public Health, Southeast University, Nanjing, China

**Keywords:** myopia, prevalence trend, interventions, children and adolescents, Eastern China

## Abstract

**Purpose:**

First, to investigate the utilization rate and effect of proven myopic interventions. Second, to predict the prevalence of myopia and high myopia, as well as Years Lived with Disability (YLD) caused by an uncorrected refractive error in children and teens in Eastern China from 2010 to 2050 under different interventions.

**Methods:**

(1) The surveillance of common diseases among children and adolescents in Jiangsu Province from 2010 to 2021 provides the database for myopia screening and intervention utilization surveys. (2) The National Bureau of Statistics and the Global Burden of Disease Study 2016 (GBD2016) are the foundation for the estimated myopes and YLD. (3) A systematic review provides the strong or weak impact of intervention in the prediction model. (4) The trend of screening myopia from 2010 to 2050 under various treatments is predicted using a GM (1,1) model.

**Results:**

By the year 2050, myopia is expected to affect 8,568,305 (7–12 years old) and 15,766,863 (13–18 years old) children and adolescents, respectively (95% CI: 8,398,977–8,737,633). The utilization prevalence of myopia-proven interventions for myopic children included outdoor activities, orthokeratology lenses, atropine treatment, contact lenses, frame glasses, and eye exercises, with respective rates of 31.9–33.1, 2.1–2.3, 6.0–7.5, 2.2–2.7, 60.4–62.2, and 64.7–72.5%. All interventions have substantial effects on myopia after parental myopia and behavior pattern adjustment, including physical activity, near work, dietary pattern, and sleep. Under strong intervention, the estimated reduced myopia prevalence by the year 2050 is 1,259,086 (95% CI: 1,089,758–1,428,414) for children aged 7–12, and 584,785 (95% CI: 562,748–606,823) for children aged 13–18, respectively.

**Conclusion:**

Among myopic Chinese children and adolescents, the use rates and effects of proven myopia interventions vary. Under the present intervention strategy, the prevalence of myopia and high myopia will increase from 2010 to 2050. The overall number of myopic people can be greatly decreased by implementing timely, steady, comprehensive interventions.

## Introduction

Myopia occurs because the cornea or lens is too powerful or because the eyeball is longer than normal (1). Uncorrected refractive error is the most common cause of distance vision impairment and the second most common cause of blindness (2). In myopia, distant objects are focused in front of the retina instead of on it, as occurs in non-myopic individuals. Holden estimated that by 2050, there will be 4,758 million people with myopia (49.8% of the world population) and 938 million people with high myopia (9.8% of the world population) (3).

The prevalence of myopia is severe in China, and visual impairment occurs with complications of high myopia, such as retinal detachment, cataracts, glaucoma, and blindness (4–7). Anhui province, Fujian province, Jiangsu province, Jiangxi province, Shandong province, Shanghai city, and Zhejiang province are the seven provinces that make up the eastern mainland of China (also known as Eastern China). An area of 798,300 km^2^ in Eastern China is home to approximately 30% of the country’s population and 40% of its GDP (8). The prevalence of myopia and vision impairment is high among school students in Eastern China, and the greater prevalence rate of myopia in Eastern China is significantly influenced by the increased academic load on students at younger ages (5, 9, 10).

The effects of myopia interventions on children and adolescents have been extensively studied worldwide. Pharmaceuticals, optical devices, and lifestyle changes are among the interventions that had proven effects (11). Increasing the amount of time that children spent outdoors at school resulted in statistically significant reductions in incident myopia and myopic shift, as shown by the previous studies (12). A study of 571 students aged 7–11 years in Taiwan reported a 1-year reduction in the incidence rate of myopia of 8.4% in the intervention group vs. 17.7% in the control group (13). In most cases, wearing quality eyeglasses at the correct time and properly could easily correct children’s vision problems (14). Recent surveys conducted in rural China indicate that among myopic students, less than one-third of myopic students reported the use of glasses, and more than two-third of myopic students denied wearing them. The mean (SD) spherical equivalent refractive error of participants was −2.16 (1.12) D (range, −0.625 to −4.0 D) in the right eye. In three schools, the proportion of children with myopia (both eyes’ spherical equivalent ≤ −0.5 D) ranged from 25 to 58%, whereas the proportion wearing glasses at the time of examination was between 8 and 30% (15). According to the results of a study by Yi et al., only one-sixth of myopic students in rural China used eyeglasses (16). Orthokeratology was becoming more and more popular especially in the Asia-Pacific region to control the progression of myopia in young children (17). Orthokeratology is defined as the “reduction, modification, or elimination of a refractive error by programmed application of contact lenses (18).” Also, previous reviews, meta-analyses, and clinical trials suggested that atropine eye drops conferred the best efficacy among all myopia prevention methods (19, 20).

The two main goals of this investigation are as follows: First, based on a repeated cross-sectional survey in 2019, 2020, and 2021, to examine the usage rate of myopia interventions (such as outdoor activities, orthokeratology lenses, atropine treatment, contact lenses, frame glasses, and eye exercises) among an annual random sampling of Chinese children and adolescents. To calculate the odd-ratio effects of interventions that have been modified for parental myopia and behaviors (such as physical activity, near work, dietary pattern, and sleep). Second, to predict the estimated myopia population, high myopia population, reduced myopia population, and Years Lived with Disability (YLD) of refraction and accommodation disorders (such as severe vision impairment due to uncorrected refractive error, moderate vision impairment due to uncorrected refractive error, and blindness due to uncorrected refractive error) under various intervention strategies (current intervention situation, strong intervention, and weak intervention) among children and adolescents from 2010 to 2050 in Eastern China.

## Materials and methods

### Profile of Eastern China

With seven provinces and cities, including Shanghai, Shandong, Jiangsu, Anhui, Jiangxi, Zhejiang, and Fujian, East China is one of the most developed regions in China (Supplementary Figure 1). According to a 2010 National Bureau of Statistics report, this region is home to approximately 400 million people or almost one-third of all Chinese citizens. Supplementary Figure 2 provides comprehensive data for children aged 7–18 years.

### Definition

*Definition of screening Myopia* (21, 22): (1) For children aged 7–12 years, the screening myopia is defined as uncorrected visual acuity (UCVA) < 0.5, as well as non-cycloplegic auto refraction (NCAR) < −0.50 D. (2) For children aged 13–18 years, the screening myopia is defined as UCVA < 0.5.

*Years Lived with Disability*: Long-term disability due to a given cause including blindness, moderate vision impairment and severe vision impairment (23).

*Gray model GM (1,1)* (24): The model is established based on the Gray System Theory using a time-series prediction realm. The Gray prediction model includes the classic univariate gray prediction model [GM (1,1) model] and the multi-variable gray prediction model [GM (1,N) model]. A group of new data series with the obvious trend is generated by accumulating a certain original data series, and the growth trend of the new data series is used to establish a model for prediction, and then the reverse calculation is performed by accumulating the new data series to recover the original data series, and finally, the prediction results are obtained. GM (1,1) equation:


x∧(k+1)0=x∧(k+1)1-x∧(k)1

$$\;=\left(1-e^{\overset{\wedge }{a}}\right)\left[x_{0}\left(1\right)-\frac{\overset{\wedge }{u}}{\overset{\wedge }{a}}\right]e^{-\overset{\wedge }{a}k}$$

### Physical examination measurements

Myopia screening: In Jiangsu Province, a myopia screening was carried out since 2010. With no cycloplegia, an auto-refractor (Topcon KR-800; Topcon Co., Tokyo, Japan) was used. Children without myopia and the absence of any other major eye conditions, Chinese Han students, and parents or guardians who could give informed consent were the inclusion criteria for our subjects.

### Utilization and effectiveness of interventions

Students were requested to complete a questionnaire about myopic information during 2019, 2020, and 2021 as part of an intervention usage study. **Generalized linear model (GLM) regression analysis:** GLM analysis is performed, and a log odds ratio with 95% CI is computed to assess the effect of intervention adjusted by parental myopia, physical activity, near work, dietary pattern, and sleep, and dependent variable are classified as non-myopia (P_1_), low myopia (SE more than −3.00D,P_2_), medium myopia (SE more than −6.00D,P_3_), and high myopia (SE less than −6.00D,P_4_). The regression equation is listed as follows:


$$\begin{array}{l} GLM_{\left(P1,P2,P3,P4\right)}=\beta_{0}+\beta_{main-effect1(Intervention1)}X_{1}\\ \;\;+\beta_{main-effect2(Intervention2)}X_{2}+\beta_{main-effect3(Intervention3)}X_{3}\\ \;\;+\beta_{main-effect4(Intervention4)}X_{4}+\beta_{main-effect5(Intervention5)}X_{5}\\ \;\;+\beta_{main-effect6(Intervention6)}X_{6}+\beta_{adjusted-parentmyopia}X_{7}\\ \;\;+\beta_{adjusted-physicalactivity}X_{8}+\beta_{adjusted-nearwork}X_{9}\\ \;\;+\beta_{adjusted-dietarypattern}X_{10}+\beta_{adjusted-sleep}X_{11} \end{array}$$

### GM (1,1) model predicting the trend of screening myopia from 2010 to 2050 under current interventions

Data are used from 2010 to 2021 as the original data to establish a GM (1, 1) model (25) and to predict the number of myopic students from 2022 to 2050. **Related parameters are obtained from:** (1) surveillance of common diseases among students in Jiangsu Province from 2010 to 2021; (2) Global Burden of Disease Study 2016 (GBD2016) (26) and National Bureau of Statistics; and (3) a systematical review. Details on these parameters can be found in Supplementary Table 1. In this study, we also predict a reduced myopia population under weak intervention (R: 0 to 0.25 D/year, more outdoor activities, MOA, 0.14 D/year) and strong weak intervention [R: >0.50 D/year, high-dose atropine (1 or 0.5%), ATRH, 0.7 D/year] (27). (4) Projected demographic changes in the relevant age groups are shown in Supplementary Figure 2.

### Statistical analysis

Continuous variable trend analysis is a one-way variance trend analysis, with mean ± SD as the description. For discontinuous variables, chi-square is used for trend analysis and percentage as the description. Indicators including weight, height, vision, and spherical equivalent (SE) were standardized using age. The standardization makes the proportion of the population per age as 1:1.

All data are analyzed using office software and SPSS V.20.0 software.

### Ethics statement

The Institutional Review Board approved the Ethics Committee of Jiangsu Province CDC’s study protocol. The students and their parents are informed about the survey’s aim, and teachers obtained participants’ and their parents’ oral and written consent. Detailed information can be found in the previous article (28–30).

## Results

### Characteristics of the study participants from 2010 to 2021

**FIGURE 1:**
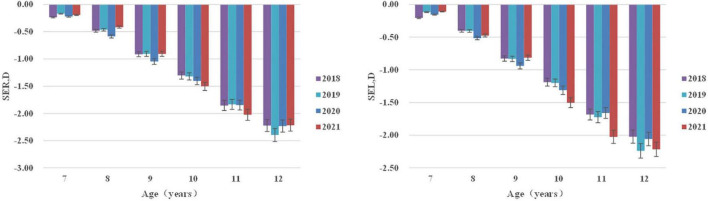
Spherical equivalent (SE, D) distribution of right/left eyes stratified by age from 2018 to 2021.

A total of 563,185 students participated in this study from 2010 to 2021. The adjusted distribution of sex is not significant (*P* > 0.05). Age-standardized weight and height show upward trends, while age-standardized vision shows downward trends (trend *P* < 0.01) (Table 1). Changes in spherical equivalent (SE, D) from 2018 to 2021 are not significant (trend *P* > 0.05) (Figure 1).

**TABLE 1:** The demographic characteristics of the study participants from 2010 to 2021 aged 7–18 years.

	Number of survey population	Male/Female (*P* = 0.223)	Age-standardized height (cm) (*P* < 0.001)		Age-standardized weight (kg) (*P* < 0.001)		Age-standardized vision (*P* < 0.001)	
**Year**	**7–18**	**7–18**	**7–12**	**13–18**	**7–12**	**13–18**	**7–12**	**13–18**
2010	12,000	50.0/50.0	141.5 ± 11.8	165.7 ± 8.3	35.4 ± 10.3	58.7 ± 12.2	4.81 ± 0.29	4.54 ± 0.39
2011	12,000	50.0/50.0	141.6 ± 12.3	166.0 ± 8.5	36.8 ± 11.0	57.1 ± 11.8	4.89 ± 0.30	4.48 ± 0.38
2012	48,295	52.4/47.6	146.6 ± 15.0	165.4 ± 8.7	40.3 ± 13.5	57.7 ± 12.4	4.81 ± 0.38	4.44 ± 0.39
2013	57,500	52.6/47.4	141.2 ± 13.1	166.2 ± 9.1	36.7 ± 11.9	57.9 ± 12.9	4.72 ± 1.15	4.35 ± 0.90
2014	71,650	53.5/46.5	141.5 ± 12.4	166.5 ± 8.5	37.6 ± 11.7	58.4 ± 12.4	4.84 ± 0.31	4.52 ± 0.36
2015	73,687	52.6/47.4	140.7 ± 12.1	165.9 ± 8.7	36.4 ± 11.2	57.8 ± 12.0	4.88 ± 0.29	4.50 ± 0.36
2016	60,857	53.7/46.3	143.7 ± 10.6	166.2 ± 8.6	40.3 ± 9.8	58.3 ± 12.0	4.86 ± 0.29	4.48 ± 0.35
2017	30,067	50.9/49.1	141.4 ± 12.2	165.2 ± 8.5	37.9 ± 12.0	59.9 ± 12.8	4.85 ± 0.30	4.47 ± 0.38
2018	52,694	53.1/46.9	141.1 ± 12.4	165.3 ± 8.8	38.3 ± 12.5	60.2 ± 13.7	4.79 ± 0.32	4.39 ± 0.40
2019	48,649	52.7/47.3	141.8 ± 12.5	166.2 ± 8.4	38.1 ± 12.8	61.3 ± 14.3	4.79 ± 0.33	4.41 ± 0.39
2020	48,288	52.7/47.3	142.0 ± 12.6	166.3 ± 8.4	39.3 ± 13.2	62.0 ± 14.3	4.78 ± 0.31	4.41 ± 0.38
2021	47,498	52.4/47.6	141.9 ± 12.6	166.4 ± 8.3	39.2 ± 13.0	62.9 ± 14.9	4.76 ± 0.33	4.42 ± 0.38

### Current myopic intervention situation

**FIGURE 2:**
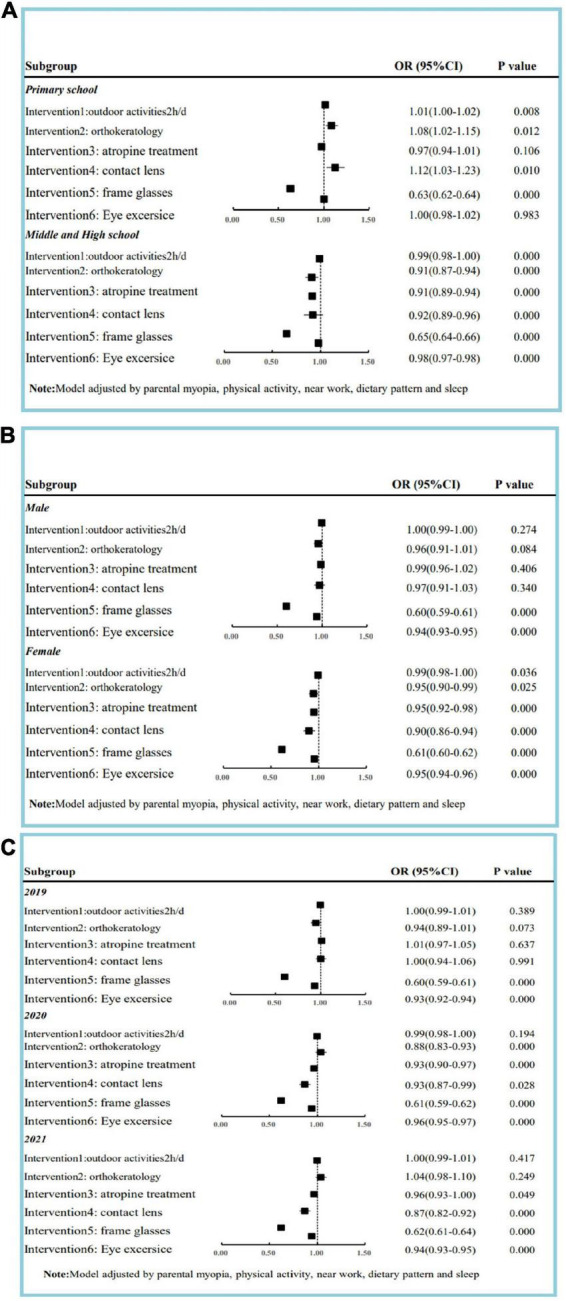
Effect of intervention on myopia adjusted by parental myopia and behavior pattern among children and adolescents based on survey in 2019, 2020, and 2021. **(A)** Effect of intervention on myopia classified by study period. **(B)** Effect of intervention on myopia classified by gender. **(C)** Effect of intervention on myopia classified by year.

The utilization rate of interventions (outdoor activities/atropine treatment) reveals a decreasing trend from 2019 to 2021 (trend *P* < 0.01). Orthokeratology has the lowest utilization rate (2.1–2.3%) and eye exercises the highest (64.7–72.5%) (Table 2). Effects of all interventions on myopia adjusted by parental myopia and behavior pattern (including physical activity, near work, dietary pattern, and sleep) are significant. The odds ratio value for frame glass of <0.70 indicates a medium effect on the progression of myopia. While orthokeratology, atropine treatment, and contact lenses took up a relatively low utilization rate; protective effects on myopia could still be observed. Outdoor activities and eye exercise showed a weak protective effect on primary and middle/high school students, respectively (Figure 2).

**TABLE 2:** Current myopic intervention utilization among myopic Chinese children and adolescents based on surveys from 2019, 2020, and 2021.

	2019	2020	2021	Absolute increase	*P-*value for trend
**Intervention 1: Outdoor activities > 2 h/d**					
Primary/middle and high school	38.7/31.3	39.4/30.7	39.0/29.4	−1.2	0.003
Male/female	37.0/29.1	36.1/29.5	35.3/28.5		
Urban/rural	34.0/31.8	32.8/33.0	32.4/31.3		
	33.1	32.9	31.9		
**Intervention 2: Orthokeratology**					
Primary/middle and high school	2.5/2.0	2.3/2.2	2.6/2.2	0.2	0.227
Male/female	2.1/2.1	2.2/2.2	2.3/2.2		
Urban/rural	2.2/2.0	2.5/1.8	2.6/1.8		
	2.1	2.2	2.3		
**Intervention 3: Atropine treatment**					
Primary/middle and high school	10.2/5.9	10.7/6.7	9.4/5.1	−0.8	0.001
Male/female	6.6/7.0	7.2/7.9	6.0/6.0		
Urban/rural	6.6/7.0	7.8/7.2	6.4/5.5		
	6.8	7.5	6.0		
**Intervention 4: Contact lens**					
Primary/middle and high school	1.5/3.0	1.2/2.5	1.0/2.9	−0.2	0.114
Male/female	1.8/3.6	1.4/3.1	1.5/3.5		
Urban/rural	3.3/1.8	2.7/1.6	3.1/1.6		
	2.7	2.2	2.5		
**Intervention 5: Frame glasses**					
Primary/middle and high school	40.9/65.3	44.2/66.9	45.1/67.1	1.7	0.000
Male/female	56.3/64.5	57.4/67.1	57.7/66.5		
Urban/rural	61.7/58.4	63.3/60.6	63.5/60.1		
	60.4	62.2	62.1		
**Intervention 6: Eye exercise**					
Primary/middle and high school	94.9/65.4	88.3/56.7	95.2/64.3	0.0	0.941
Male/female	71.9/73.2	64.7/64.7	72.0/73.0		
Urban/rural	66.7/81.1	58.0/74.7	65.6/82.4		
	72.5	64.7	72.5		

### Estimated population of myopia, high myopia, and YLD from 2010 to 2050 under the current situation

**FIGURE 3:**
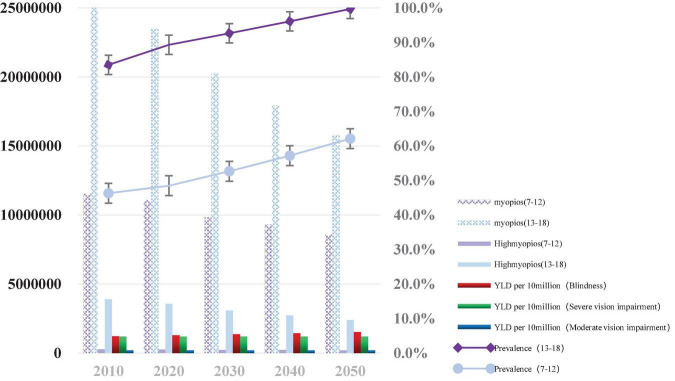
Graph depicting the estimated number of children and adolescents with myopia, high myopia, and Years Lived with Disability (YLD) from 2010 to 2050 under the current intervention situation. *YLD per 10 million (Blindness: Blindness due to uncorrected refractive error per 10 million children and adolescents); YLD per 10 million (Moderate vision impairment: Moderate vision impairment due to uncorrected refractive error per 10 million children and adolescents); YLD per 10 million (Severe vision impairment: Severe vision impairment due to uncorrected refractive error per 10 million children and adolescents).

By the year 2050, the estimated population of myopia will be 8,568,305 (95% CI: 8,398,977–8,737,633) for 7–12 years old and 15,766,863 (95% CI: 15,744,826–15,788,900) for 13–18 years old, respectively. The number of high myopia cases is 205,639 (95% CI: 201,575–209,703) for 7–12 years old and 2,395,613 (95% CI: 2,392,264–2,398,961) for 13–18 years old, respectively. The prevalence of myopia will be 62.1% for 7–12 years old and 99.7% for 13–18 years old, respectively. The prevalence of high myopia will be 1.5% for 7–12 years old and 15.2% for 13–18 years old, respectively. The estimated YLD/1,000 person-year (blindness due to uncorrected refractive error) is 151.3 (95% CI: 100.3–210.4) among children and adolescents (Figure 3).

Internal validation: The regressive performance of the GM (1,1) model is presented in Supplementary Figure 3, and the *R*^2^ values are >0.900.

### Estimation of the reduced number of the myopes with strong/weak intervention

**FIGURE 4:**
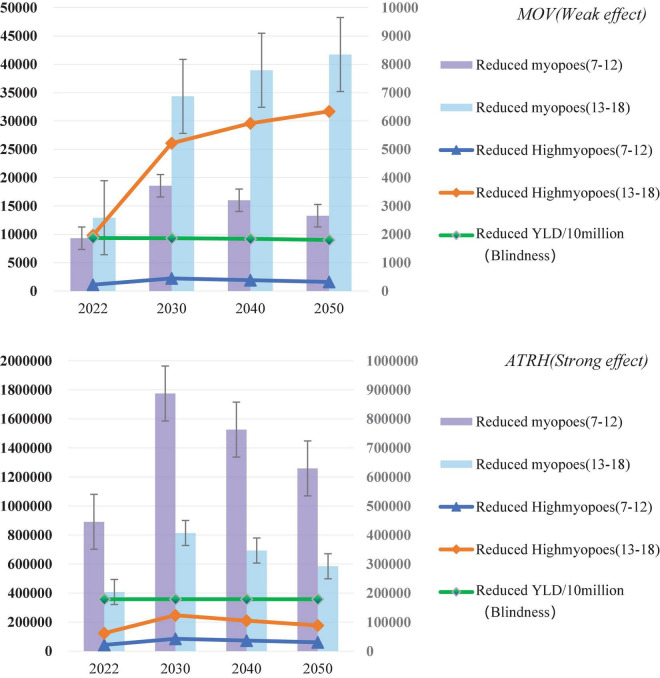
Estimates for 2022–2050 after MOV (weak effect) and ATRH (strong effect) are 100% covered: the number of myopia reductions, the number of high myopia reductions, and the rate of Years Lived with Disability (YLD) (blindness) reductions.

**(1) Weak effect intervention (MOV)**: By the year 2050, the estimated reduced population of myopia is 13,295 (95% CI: 0–182,623) for 7–12 years old and 41,731 (95% CI: 19,694–63,768) for 13–18 years old, respectively. For 7–12 years old, the reduced population of high myopia was 319 (95% CI: 0–4,383) and for 13–18 years old, it was 6,341 (95% CI: 2,992–9,689). **(2) Strong effect intervention (ATRH)**: The estimated reduced population of myopia by 2050 is 1,259,086 (95% CI: 1,089,758–1,428,414) for 7–12 years old and 584,785 (95% CI: 562,748–606,823) for 13–18 years old. The population of high myopia is 30,218 (95% CI: 26,154–34,282) for 7–12 years old and 88,852 (95% CI: 85,504–92,200) for 13–18 years old, respectively (Figure 4).

## Discussion

According to our study, there will be roughly 24.3 and 2.6 million children and adolescents in Eastern China between the ages of 7 and 18 who have myopia or high myopia, respectively. Among myopic Chinese children and adolescents, varying percentages of effective myopia interventions (such as orthokeratology lenses, atropine therapy, contacts, glasses frames, and outdoor activities) were used. By 2050, myopia and high myopia are predicted to be reduced by nearly 55,027–1,843,872 and 6,660–119,070, respectively, according to estimates of weak or strong intervention effects. These have essential implications for myopia interventions, such as population-level and personal preventive strategies.

What are the main factors affecting our projects? First, lifestyle factors such as physical activity and close work have significantly impacted the onset and progression of myopia (31). High-pressure educational systems in Eastern China can be listed as a crucial factor (32). The human environment in this area supports culture and fosters education, and since ancient times education has received significant attention in this region. For instance, Confucius, who was born in the Eastern Chinese province of Shandong, is revered as a representative of Chinese culture and thinkers. Myopia growth may be influenced by “culture-gene.” Jiangsu Province has been considered a representative area in Eastern China from the perspective of economic, educational, and cultural levels. Economic, educational, and cultural levels are highly correlated with myopia. Jiangsu Province is used in this study to approximate the myopia level in Eastern China. Japan, Singapore, and Hong Kong (China), which are part of the “Circle of Confucius-Culture” and share a lot of the same values, social structures, and cultural traditions, may be also impacted by this phenomenon.

Second, the effect of myopia controls the interventions. The prevalence of myopia is rapidly increasing globally, and this phenomenon has driven investment into myopia prevention and control (33). In this study, interventions include exhibited significant effects after adjusting parental myopia and behavior patterns among students sorted by age and gender. However, utilization rates of proven myopic interventions are still relatively low. Interventions that sufficiently slow down or delay myopia can potentially prevent an individual from developing high myopia provided treatment is started early enough (3). We estimated the reduced number of myopia, high myopia, and related YLD after strong/weak interventions. This indicated that a concerted effort by the government, education, and health systems should be undertaken to control myopia.

Different interventions have been attempted to reduce myopic progression, including increasing outdoor time (12), optical methods [orthokeratology (34, 35), contact lens (36, 37), and eyeglasses], and pharmacological methods including atropine eye drops (38). Spending 10–14 h per week in outdoor activities (MOA) as compared to “engaging in outdoor activities” only 0–5 h per week was associated with approximately half the risk of developing myopia (39). Atropine treatment shared a relatively lower usage rate in Eastern China. High-dose atropine (0.5–1%) is the most effective, but it has significant trade-offs with respect to the rebound of myopia on discontinuation and side effects. Low doses of atropine have been trialed and show a dose-dependent efficacy (40). Students receiving interventions (orthokeratology lenses, atropine treatment, contact lenses, and frame glasses) were more likely to have a higher level of myopia than those not receiving interventions (Supplementary Table 2). As explained in the introduction section, due to specific differences in the cognitive level of myopia, parents pay more attention to myopia intervention for children with high myopia. In contrast, parents ignore children with low myopia, leading to such a phenomenon in single-factor analysis. In the multivariate analysis, the addition of correction variables reduces the occurrence of such bias. Age, gender, level of myopia, family type, parent, region economic level, and active medical treatment record significantly impacted intervention utilization among Chinese children (Supplementary Table 3).

Our study design has some potential limitations. (1) The population predicted comes from the general education group, and the population’s myopia from education diversion is not considered. (2) The definition of screening myopia was based on our previous studies; in 2018, we randomly selected 36 primary and secondary schools in 12 counties of Jiangsu Province and conducted myopia diagnosis and myopia screening for 7,441 students aged 7–18 years. We suggested that under the condition of non-mydriasis and large populations, we recommend that the myopia screening strategy for students aged 7–12 was UCVA < 0.5 and NCAR < −0.50D, and the myopia screening strategy for students aged 13–18 was UCVA < 0.5. Myopia screening data are utilized, but changes in optical refraction before and after cyclic events are not considered. This could lead to an overestimation of myopic individuals in future. (3) Frame glasses and contact lenses, such as single and defocus, with different myopia control effects, were not differentiated in the study, which may yield bias on utilization and effect of intervention estimation.

Strengths can be listed as follows: (1) From 2010 to 2022, Eastern China’s ongoing large-sample size surveillance of myopia screening can correctly describe the health trends of children and adolescents. (2) Considering the influence of myopia control interventions and a thorough prediction of the decrease in myopes among children and adolescents, this study provides a research foundation for the burden of myopia disease in Eastern China.

In conclusion, our research offers predictions for myopia, high myopia, and YLD of refraction and accommodation disorders through various interventions by 2050. The usage and effects of myopia interventions (such as outdoor activities, orthokeratology lenses, atropine treatment, contact lenses, frame glasses, and eye exercises) vary among Chinese children and adolescents. The total number of children and adolescents with myopia in East China declined from 2010 to 2050 under the current intervention status, while the prevalence of myopia significantly increased. Myopia prevalence can be significantly decreased by increasing strong and weak interventions. A concerted campaign by the government, schools, and health systems should be made to prevent myopia, as Eastern China has long placed a high value on education.

## Data availability statement

The original contributions presented in this study are included in the article/Supplementary material, further inquiries can be directed to the corresponding authors.

## Ethics statement

The studies involving human participants were reviewed and approved by Ethics Committee of Jiangsu Province CDC. Written informed consent to participate in this study was provided by the participants’ legal guardian/next of kin.

## Author contributions

XZ and JY: data curation. YW and YZ: investigation and methodology. WD: project administration and supervision. XZ: design, advice, and writing the original draft. WD and YZ: manuscript modification. All authors have read and approved the manuscript.
