# Supplementary material for: Trend of myopia through different interventions from 2010 to 2050: Findings from Eastern Chinese student surveillance study
Source: Front Med (Lausanne). 2023 Jan 18;9:1069649. doi: 10.3389/fmed.2022.1069649 (PMC9889364; doi:10.3389/fmed.2022.1069649)
Supplement: Supplementary file 1 [file Data_Sheet_1.docx]

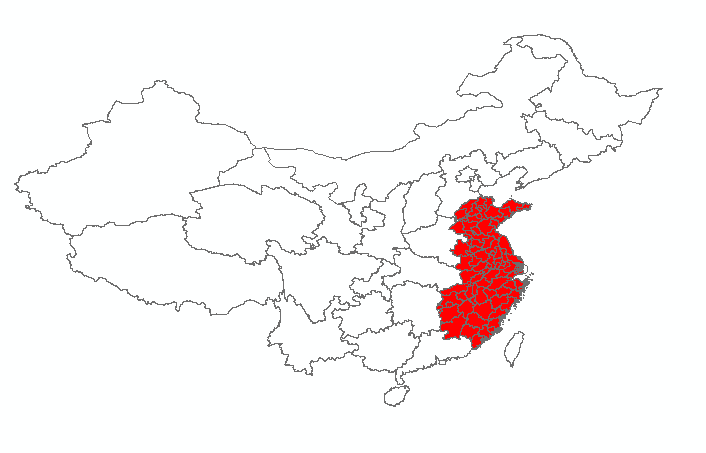


Supplement Figure1 Location of Eastern China

Supplement Figure2 Estimated eastern China population aged 6-18 years from 2000 to 2050

Supplement figure3 Prediction and observed values of GM (1,1) model

Supplement Table1 Parameters used in GM (1,1) for projections

| Interpretation | Parameter (Formula) | Estimated | Reference |
| --- | --- | --- | --- |
| Distribution of population aged 7-18 in China | G | Year 2050:  7~12: 13794685  13~15: 8545750  16~18: 7269219 | National Bureau of Statistics |
| Ratio of population in East China | α |  |  |
| Estimated East China population (7-18 years old) | B=G×α |  |  |
| Blindness due to uncorrected refractive error YLD Disability weight | DW_1_ | 0.187(0.124–0.26) | Global Burden of Disease Study 2016 (GBD 2016) Data Resources |
| Severe vision impairment due to uncorrected refractive error Disability weight | DW_2_ | 0.184(0.125–0.258) |  |
| Moderate vision impairment due to uncorrected refractive error Disability weight | DW_3_ | 0.031(0.019–0.049) |  |
| More outdoor activities (MOA) | I_1_ | 0.14 (0.06-0.22) D/year | Huang J, Wen D, Wang Q, et al. Efficacy Comparison of 16 Interventions for Myopia Control in Children: A Network Meta-analysis. Ophthalmology. 2016;123(4):697-708. |
| High-dose atropine (1% or 0.5%) (ATR H) | I_2_ | 0.7 (0.5-0.8)D/year |  |
| MOA Utilization rate | B_1_ | 31.9%~33.1% | JS Survey |
| ATRH Utilization rate | B_2_ | 6.0%~7.5% |  |
| Effect of MOA | I_1_×(1-B_1_) | 0.095(0.041-0.150)/year |  |
| Effect of ATRH | I_2_×(1-B_2_) | 0.66(0.47-0.75) D/year |  |
| Prevalence of myopia from  2010 to 2021 | M | 7~12:46.3%~52.6%  13~18:83.5%~89.5% |  |
| Proportion of high myopia | PHM | 7~12:2.4%  13~18:15.2% |  |

Supplement Table2 Comparison of spherical equivalent between intervention received and Not received groups among myopic Chinese children and adolescents aged 10 to 15 years from 2019-2021

|  |  | SER | P | SEL | P |
| --- | --- | --- | --- | --- | --- |
| Intervention1:outdoor activities>2h/d | Received | -3.12±0.01 | 0.00 | -3.08±0.10 | 0.51 |
|  | Not received | -3.39±0.07 |  | -3.17±0.07 |  |
| Intervention2: orthokeratology | Received | -5.96±1.68 | 0.00 | -5.80±1.68 | 0.00 |
|  | Not received | -3.41±0.03 |  | -3.22±0.05 |  |
| Intervention3: atropine treatment | Received | -3.62±0.04 | 0.02 | -3.44±0.04 | 0.03 |
|  | Not received | -3.46±0.06 |  | -3.27±0.07 |  |
| Intervention4: contact lens | Received | -3.91±.078 | 0.00 | -3.69±0.08 | 0.00 |
|  | Not received | -3.47±0.06 |  | -3.28±0.06 |  |
| Intervention5: frame glasses | Received | -3.91±0.04 | 0.00 | -3.69±0.04 | 0.00 |
|  | Not received | -2.83±0.12 |  | -2.68±0.15 |  |

Supplement Table3 Associated factors on the willing to receive interventions among myopic Chinese children and adolescent

|  | Intervention1:outdoor activities>2h/d  OR(95%CI) | Intervention2: orthokeratology  OR(95%CI) | Intervention3: atropine treatment  OR(95%CI) | | Intervention4: contact lens  OR(95%CI) | Intervention5: frame glasses  OR(95%CI) |
| --- | --- | --- | --- | --- | --- | --- |
| Basic information Age | 1.105  (1.088-1.122) | 0.946  (0.897-0.998) | 1.084  (1.051-1.118) | 0.762  (.715-.812) | | 0.828  (0.814-0.843) |
| Gender | 1.402  (1.351-1.456) | - | - | 0.665  (0.563-0.787) | | 0.595  (0.571-0.621) |
| Level of myopia | 1.041  (1.009-1.074) | 0.695  (0.631-0.765) | 0.759  (0.717-0.804) | 0.794  (0.708-0.890) | | 0.409  (0.391-0.429) |
| Height | 0.996  (0.994-0.999) | - | 1.008  (1.003-1.013) | - | | 0.990  (0.987-0.993) |
| Family type  Extended | 0.930  (0.885-0.977) | - | - | 1.398  (1.158-1.688) | | 0.881  (0.833-0.931) |
| Nuclear | - | 0.681  (0.565-0.821) | - | 1.683  (1.399-2.023) | | 0.880  (0.835-0.929) |
| Parent  Father education(＞high school) | - | 0.646  (0.568-0.735) | 0.829  (0.767-0.895) | - | | 0.938  (0.897-0.981) |
| Mother education(＞high school) | - | 0.595  (0.524-0.675) | 0.855  (0.794-0.920) | - | | 0.925  (0.887-0.964) |
| Parental myopia | - | 1.075  (1.018-1.135) | - | - | | 1.068  (1.049-1.086) |
| Region economic level(low-high) | 1.155  (1.115-1.198) | - | 1.082  (1.006-1.163) | 1.276  (1.096-1.484) | | - |
| Active medical treatment(＞4/y) | 0.867  (0.852-0.881) | 0.601  (0.566-0.637) | 0.828  (0.801-0.856) | 0.827  (0.771-0.887) | | 0.834  (0.818-0.850) |
